# Eliciting empathetic drives to prosocial behavior during stressful events

**DOI:** 10.3389/fpsyg.2022.963544

**Published:** 2022-10-20

**Authors:** Nicola Grignoli, Chiara Filipponi, Serena Petrocchi

**Affiliations:** ^1^Cantonal Socio-Psychiatric Organisation, Public Health Division, Department of Health and Social Care, Repubblica e Cantone Ticino, Mendrisio, Switzerland; ^2^Department of Internal Medicine, Regional Hospital of Bellinzona and Valleys, Ente Ospedaliero Cantonale, Bellinzona and Università della Svizzera italiana, Lugano, Switzerland; ^3^Department of Oncology and Hemato-Oncology, University of Milan, Milan, Italy; ^4^Applied Research Division for Cognitive and Psychological Science, IEO European Institute of Oncology IRCCS, Milan, Italy; ^5^Faculty of Biomedical Sciences, Università della Svizzera italiana, Lugano, Switzerland; ^6^Lab of Applied Psychology and Intervention, Università del Salento, Lecce, Italy

**Keywords:** empathy, prosocial behavior, stress, social intervention, fiction, theatre, COVID-19, pandemic

## Abstract

In the current pandemic situation, psychological science is increasingly considered by public health policy. Empathy is mainly recognized as a crucial drive for prosocial behavior. However, this rich body of evidence still lacks visibility and implementation. Effective social programs are needed, and little is known about how to elicit empathetic drives. The paper gives first a clear foundation to the role of empathy during stressful events. It provides then a comprehensive overview of innovative interventions triggering empathic response in the public such as fiction, film, and theater. Moreover, it integrates interactive ways of sharing personal views that could elicit empathetic feelings in different people. Advances deriving from this perspective could be of significant public interest in the current and future health crises and help authorities develop innovative social programs, which should be the focus of further scientific inquiry.

## Introduction

During the COVID-19 pandemic, psychological science stood at the forefront for guiding behavioral containment of the contagion (Habersaat et al., 2020) and informing global health policy (Betsch et al., 2020; Van Bavel et al., 2020). Public health measures were accompanied by specific and evidence-based health communication strategies (Ratzan et al., 2020), concentrating on scientific information to promote protective behaviors (Fagerlin et al., 2010). Besides personal risk-management and self-protection strategies, prosocial attitudes play a pivotal role in adherence to public health measures (social distancing, self-isolation, and vaccination; Habersaat et al., 2020). During an emergency outbreak, clear, reliable, and accessible measures have to be provided, allowing people to understand the situation and adhere freely to the public health policy (Brooks et al., 2020). If cognitive information processing was immediately considered fundamental, the central role of emotions in personal and collective behavior is increasingly acknowledged (Coifman et al., 2021). Empathy has gained the spotlight as a powerful drive for prosocial behavior (Bellato, 2020; Christner et al., 2020; Pfattheicher et al., 2020; Petrocchi et al., 2021; Karnaze et al., 2022). Prosocial behavior is intended as the help given to others, not motivated by professional or organizational duties. It could be aimed at personal interests such as obtaining social approval or reducing one’s own distress and should be differentiated from altruism, which seeks to benefit the other person (Bierhoff, 2002). Empathy could be seen as an innate ability to perceive and be sensitive to the emotional states of others that can drive prosocial behavior. It can be elicited by mental processes like those activated by imagination or reading fiction (Decety et al., 2016). Still, we should be aware of the risk of oversimplifying the different psychological components underpinned by this concept. Therefore, it seems necessary to define the empathic processes implicated in prosocial behavior and sustain them through adequate public health policy.

A decline in adherence to COVID-19 protective behaviors has been observed in the general population, which can be explained through a combined effect of the economic burden, modulation of risk perception, varying public enforcement measures, and motivational issues due to chronic stress exposure (Petherick et al., 2021). Healthcare professionals show signs of exhaustion and compassion fatigue (Lluch-Sanz et al., 2022). However, besides some isolated examples of “social prescribing” (namely, non-medical interventions in public health; Drinkwater et al., 2019; Hossain et al., 2020; Fixsen et al., 2021), authorities seem to have failed to find effective interventions for maintaining prosocial behavior during this or future health emergencies. To achieve this goal, the paper introduces the reader to the role of empathy during COVID-19, giving a theoretical explanation of how empathy could elicit prosocial behaviors and why it might work under stressful situations. The paper will then review the available literature regarding the best social strategies to produce empathetic drives. It mainly focuses on narrative, film, theater, and more immersive possibilities that new technologies offer. Participating in fiction may trigger specific components of empathy without increasing distress and seems necessary in order to maintain prosocial behavior during stressful events. Finally, this article provides insights for creating further prospective studies evaluating the effectiveness of such interventions and helping public health authorities integrate empathy into public health communication strategies and public health policy.

## Empathy’s role in the aftermath of a stressful event

### Empathy definition and implications

Empathy is a core construct extensively studied in social psychology for its influence on intergroup conflicts and prosocial behavior (Morelli et al., 2014; Decety et al., 2016; Cameron et al., 2022). According to Rogers and Kinget (2013), empathy is viewed as “perceiving the subjective world of others as if we were this person... (p. 233).” In other words, an empathetic person understands the feelings and emotions of others without identifying themself with that person but feeling the same emotional suffering “as if we are” the other. Advances in scientific psychology and neurosciences have contributed to a detailed comprehension of this general definition, including *affective sharing*, *empathic concern*, and *perspective-taking*. A functional architecture of empathy, which encompasses such different psychological components and integrates the affective, motivational, and cognitive processes, and their specific neurological correlates, has been proposed by Decety and Jackson (2004). Integrating hypotheses from social and evolutionary psychology (Batson et al., 1987; Batson, 1997) and focusing on self-awareness and emotion regulation, Decety and Jackson have mainly developed the motivational processes that make empathy a distinctive trait of human society (Decety and Jackson, 2004). Empathy resides more in caring for someone rather than the somehow naïve idea of perspective or emotional sharing, which could be seen as relevant for designing specific healthcare interventions (Decety, 2020). Indeed, the peculiarity of empathy directed to the relief of the other’s suffering (i.e., what has been called *empathic concern* or, more generally, compassion) is to decrease the risk of emotional distress, which could block, in turn, prosocial behavior. If moral choices could be seen as generated by negative emotional states that we aim to master (Prinz, 2011), affective and cognitive empathy combined with compassion could be understood as a way of reducing such discomfort. Decety et al. (2016) further affirm that helping and caring are inherently rewarding, and empathy is seen as a fundamental drive of prosocial human behavior beyond a goal-oriented or tension-reduction model that implies the concept of motivation. This is, in our perspective, relevant for prosocial behavior in the pandemic when the public aims to reduce the suffering of others independently of individual experience, social background, or group identity.

### Mechanisms of “safe” empathy during stressful events

During collective emergencies, empathy is the basis for interpersonal cooperation and is triggered in the individual by stress. Post-traumatic stress disorders are the pathological consequences of adverse events, but positive growth may follow challenging life situations (Tedeschi and Calhoun, 2004). Trauma increases a person’s attention to adverse environmental cues and negative emotions (due to amygdala responsiveness; Blair et al., 2013). This hypervigilance (or arousal) protects against negative stimuli and elicits a greater awareness of the emotional states that other people might experience if they are exposed to similar situations. This process is called emotion regulation, referring to managing their own emotions and those of others (Gross, 2015). Still, emotion regulation is a multifaceted capacity involving not only cognitive coping (rethinking what to do) but also behavioral strategies (what individuals actually do) when a person is facing adverse events (Coulacoglou and Saklofske, 2017). Empathy and emotion regulation are not distinct processes but part of the same framework. Namely, the cognitive dimension of empathy overlaps with the neural architecture of emotion regulation, and the mimicry/embodiment process operates as a hyperlink between them, eliciting an affective response (Thompson et al., 2019). Indeed, different and interacting neural networks are behind empathy’s components contributing to its experience. The sub-cortical network regulates affective arousal (i.e., amygdala, hypothalamus, orbitofrontal cortex, ventral striatum, and insula); while the cognitive dimension related to the *Theory of Mind* (ToM) is associated with cortical network activation (including the medial prefrontal cortex, the temporoparietal junction, the superior temporal sulcus, and the anterior temporal pole). Last but not least, emotion regulation (motivational component) depends on executive functions instantiated in the intrinsic connections with both cortical and subcortical structures (Chakrabarti et al., 2006; Decety, 2010; Engen and Singer, 2013; Thompson et al., 2019).

Affective, motivational, and cognitive dimensions of empathy are intrinsically linked; the difference is found in how these systems work. The cognitive dimension operates on a top-down level through attention and control over the situation and alerting the person to adverse events. The affective dimension works at a bottom-up level requiring a safe context in which the person can feel protected and supported to embody the regulation capacity offered by primary and significant reference models (Siegel, 2012; Filipponi et al., 2020). The emotion regulation process functions as a link between affective and cognitive empathy; it helps maintain cognitive and behavioral self-awareness and might represent the core to guide interventions for eliciting prosocial behaviors in healthcare (Decety, 2020). The potential risk that eliciting empathy for others’ suffering might increase distress in the population should be acknowledged. Still, the role of emotion regulation and self-awareness in the empathetic process here appears to be a convincing argument for tailoring the social intervention to control this side effect(Cionini, 2013; Fourie et al., 2017). However, empathy is socially determined, in fact, the intersubjective bond with others is fundamental, and research shows that when a person perceives the other as a “stranger” or “adversary” in a conflict, an intergroup effect may decrease the empathic response (Cionini, 2013; Vanman, 2016; Fourie et al., 2017). Moreover, other factors may modulate the experience of empathy, including features of the empathic emotion (e.g., valence, intensity, and salience), empathizer (e.g., personality, gender, etc.), the relationship with the empathizer (e.g., valuation of the other, familiarity, etc.), and so on (Engen and Singer, 2013). Bias in empathy eliciting has been found in social determinants, including outgroup and intergroup effect, prejudices and beliefs impact, and physical/face attributes (neotenous traits effect; Decety, 2021). Those biases are context dependent and should be considered with caution during stressful events because they could induce inequality in public health policy and interfere with collective decision-making. Awareness of these group-dynamics biases could help orient interventions to reduce the mechanism of prejudice. Empathy may be partially influenced by social context input but working on cognitive abilities, such as perspective taking (or ToM) and reasoning, it may allow decreasing biases and possibly influence the way in which empathy will be experienced. The top-down processes (reasoning, cognitive control, etc.) are essential for filtering and evaluating the emotional responses that guide moral decisions and decrease biases (Decety, 2021).

### Prosocial behaviors during COVID-19 elicited by empathy

Empathy has been crucial in orienting prosocial behaviors during and after the COVID-19 outbreak. For instance, Petrocchi et al. (2021) emphasized that individuals were guided by empathic concerns to protect others from COVID-19 over and above their perception of actual and potential psychological distress. In other words, empathic concern predicted the acceptance of self-isolation behavior during risk exposure regardless of depression, anxiety, and emotional distress levels. These findings have been confirmed by other studies demonstrating that not only affective and motivational empathy (Pfattheicher et al., 2020) but also the cognitive dimension (perspective-taking; Galang et al., 2021) promote willingness to adhere to COVID-19 health policies (i.e., social distancing, hand hygiene, and wearing of mask). Beside individual risk perception a main motive identified by several studies for adhering to COVID-19 restrictive measures was protecting close relationships, including vulnerable categories (e.g., individuals with chronic diseases, the elderly, etc.; Christner et al., 2020; Pfattheicher et al., 2020; Petrocchi et al., 2021). This finding was replicated by further research clarifying the role of the specific components of empathy on prosocial health behaviors. Compassion during the early pandemic outbreak was found predictive of effective prosocial behavior and perceived ability to help others independently of political ideology (Karnaze et al., 2022). Compassion is a valid predictor of prosocial behaviors even when the threat perceived by the individual does not necessarily concern them directly (e.g., pandemic scenario). For instance, Morstead et al. (2022) demonstrated that individuals who initially perceived a more significant personal threat from COVID-19 reported a greater engagement in preventive behaviors regardless of activating an empathetic response. Otherwise, in those who reported a lower perceived threat, a high association between levels of empathetic responding and the likelihood of subsequently engaging in preventive behavior was found. These results established the primary role of the motivational aspects of empathy in actual health behavior during the pandemic compared to attitudes mainly influenced by affective and cognitive empathy. A similar finding was demonstrated for the intention to get vaccinated (Pfattheicher et al., 2022), but research on how empathy may influence vaccination adherence is still ongoing.

### Is empathy long-lasting during stressful events such as the COVID-19 pandemic?

Maintaining empathy during a stressful event becomes crucial for collective solidarity and individual mental health (Sassenrath et al., 2021). Longitudinal data on empathy during stressful events and specifically during the pandemic are scarce, and demonstrations of their external validity and generalizability are few. Baiano et al. (2022) demonstrated that after 1 year of the COVID-19 outbreak, individuals showed a higher level of empathy’s cognitive (perspective-taking) and affective components. In contrast, social skills such as collaboration or active listening decreased. van de Groep et al. (2020) found contradicting results and showed a decrease in empathetic concern over time in a population of adolescents while the perspective-taking ability increased. By contrast, in generic acute stressful events (stress related to work or in daily life), a decline in empathy has been demonstrated (West et al., 2006; Park et al., 2015; Crenshaw et al., 2019) although with gender differences (i.e., in the condition of acute stress, women had significantly lower levels of empathic accuracy, but both men’s and women’s accuracy was sensitive to arousal levels; Crenshaw et al., 2019). Another study involving only healthy young men showed that the affective dimension of empathy under acute stress is detrimentally affected more than its cognitive counterpart (Wolf et al., 2015). Selective pressures shape these gender differences through human development. Specifically, females are not only stereotypically portrayed as more empathic and altruistic than males, but they are more prone to be sensitive to another’s pain or distress and reveal prosocial and other affective behaviors due to the evolutionary history of maternal care. Conversely, males showed more cognitive control and cognition when it came to cognitive empathy. This difference is consistent with the neurobiological literature indicating independence between these two systems (Christov-Moore et al., 2014). Overall, stress leads to depersonalization and emotional exhaustion (Park et al., 2015) and increases arousal (Crenshaw et al., 2019), compromising our ability to be in touch with others’ emotions, feelings, and thoughts (i.e., a state of detachment from our emotions and those of others; Morelli et al., 2015; Depow et al., 2021, Filipponi et al., 2022). Data from healthcare professionals are exciting in this regard. Indeed, a recent systematic review (Lluch-Sanz et al., 2022). has shown a rising burnout rate, dimensions of emotional exhaustion, depersonalization, and compassion fatigue in healthcare professionals after the pandemic. On the contrary, the levels of compassion satisfaction (derived by the pleasure during distressful events of helping others and providing a means to alleviate their suffering) remained stable over time. Resilience, social support, and participation in interventions to reduce burnout were identified as protective factors in this review. Data on compassion satisfaction are scarce, preliminary, and need further investigation but tend to indicate that healthcare professionals, even at risk for burnout, maintained the satisfaction of helping their patients. Therefore, it seems that the positive experience of being of help to others is a driver to keep going despite adversity.

Data outlined in this section should be considered in planning social interventions aimed at sustaining empathy during stressful events. It has been said that empathic concern can be considered the main component of empathy linked to prosocial behavior but it needs to be coupled with emotional regulation that allows control of potential distress and dynamic-group biases. In other words, compassion and self-awareness should be combined for social intervention aimed to elicit “safe” and long-term empathetic response in the public. In the next section, the reader will be guided through the best social strategies employing fiction as an innovative way of triggering such specific empathic response. Data on the use of fiction during the current pandemic situation are not yet available; the narrative review of literature will focus therefore on evidence produced by research in education, social sciences and neurosciences. The objective is to inform future studies in the emerging domain of social intervention during stressful events and to help public health authorities to integrate empathy into public health communication strategies and health policy.

## Innovative strategies to elicit empathy during stressful events

### Fiction as a root of prosocial behavior

Evolutionary theorists postulate that fiction represents a fundamental tool for social and emotional processing (Boynd, 2018). In particular, fiction is seen as a cultural way of developing self-consciousness (Lodge, 2004) and growing the necessary empathy with others for social group cooperation (McDonald, 2018). Anthropologists investigating the evolutionary origins of morality argue that language and mental ability could be seen as secondary to prosocial concerns that have emerged to allow social group practices (Burkat et al., 2018). Performing, visual and electronic arts and literature could strengthen social bonds and cohesion and change health behaviors, as confirmed by the World Health Organization (WHO) report on this topic (Faincourt, 2019). In particular, as suggested by recent cohort studies, people’s engagement in arts may act as a catalyst for prosociality and cooperation (van de Vyver and Abrams, 2018). However, the specific role of fiction in prosociality is under-recognized and could be of significant interest during stressful events. Fiction could be seen as going beyond esthetics (Fornaro, 2016) and raising the public’s awareness of citizenship’s political and moral values. The process of mimesis, the imitation of life, is the fundamental psychological process at stake in classic dramaturgy between actor and audience (de Marinis and Dwyer, 1987; Oatley, 1999). However, more recent modern dramatists such as Moreno and Boal aimed to trigger through fiction empathetic concerns that the spectator could enact to reach the objective of finding and applying creative solutions to real-life challenges (Meisiek, 2004). Moreover, contemporary dramaturgy has changed the acting codes by introducing a direct representation of life as a drama (Berghegger, 2009). Progressively, contemporary dramaturgy has become less focused on acting than on feelings by the actor and spectators who are involved in a co-construction of fictional experience. Fiction targets empathic concerns that could impact on prosocial behaviors, since the spectator will put themself in the shoes of another person thus avoiding the real experience that generates distress. Therefore the use of fiction may represent a future challenge and a suitable tool during stressful events, such as a pandemic, since it could be applied on a large-scale guaranteeing access by a huge public while respecting social norms and restrictions.

### Empathy through storytelling

In creating a profound and immersive simulation of social experience in readers, literary fiction has been described as potentially reducing social prejudice and stimulating social cognition (which entails empathy, prosociality, and other related constructs; Mar and Oatley, 2008; Koopman and Hakemulder, 2015). The evidence available to date on fiction is mainly correlational. Still, a recent meta-analysis confirms the consistency of a small positive effect on social-cognitive performance, probably enhanced by the more immersive and longer period of reading in the real world (Dodell-Feder and Tamir, 2018). Authoritative experimental works have focused mainly on the impact of reading literature on empathic competencies and demonstrated that reading fiction increases mentalizing abilities (Kidd and Castano, 2013). The fact that reading novels helps “reading minds” has been tested and verified in middle childhood (Lecce et al., 2021), but the effect of reading fiction on the ability to share emotions must still be proved. In a study using various empathy measures and pre-test post-test design, reading literary fiction appears more effective than non-fiction or science fiction in improving mentalizing abilities. Still, no discernible effect on emotion sharing has been found (Pino and Mazza, 2016). However, a recent study with two behavioral experiments on healthy adults confirms that reading experience supports emotion recognition skills overall (Schwering et al., 2021). Narrative medicine claims that putting an experience into words or reading about that experience could help cultivate empathy and improve ethical decision-making (Charon, 2001; Schneider et al., 2019). Experimental evidence of the impact of reading, or writing, of illness narrative on clinical empathy, is emerging (Lemogne et al., 2020; Schoonover et al., 2020) but needs further investigation with adequate research design. More specifically, these works suggest that reading improves cognitive and affective mentalizing abilities but that such knowledge does not influence the behavioral response to others. Recalling the previously described emotion regulation system, it could be hypothesized that reading (or writing) activates the top-down level through attention and control (cognitive empathy), eliciting indirectly an affective response that enables the reader to understand and represent the character’s emotional state. However, how the person will respond to the affect experienced may be influenced by the emotion regulation system (Thompson et al., 2019).

### Machines that generate empathy

Interestingly, if a film could be seen as *a machine that generates empathy* (James, 2014), data on the impact of cinema on empathetic growth are rare. Some cultural initiatives aim to spread empathy through film across the public domain,^1^ but evidence of this needs to be confirmed. Interest in this topic has been raised recently with novel investigation possibilities offered by neurosciences (Zak, 2015). Strikingly, neuroscientific research uses film viewing as an experimental condition for establishing brain network circuits involved in emotion processing (Raz et al., 2014, 2016). The study by Raz et al. (2014) found interesting connectivity between affective and cognitive empathy networks during intense emotional film viewing. However, the empathic reactions were context-dependent, depending on how the agent lived the empathic engagement. When the experience is perceived as a distant and objective event, the ToM processing may regulate the empathic engagement, reducing the risk of falling into maladaptive contagious reactions and allowing to “feel for” instead of “feel with”. Conversely, if the experience is lived as “we are the person who suffers” the empathic engagement will be automatically driven by embodied reactions. Indeed, empathy-related processing may be enhanced and reduced by switching between more or less cognitively regulated modes of engagement (Raz et al., 2014). Medical education is another domain of flourishing research for empathy in cinema; recent work has shown through three experiments that watching selected films has a significant but transient effect (Ahmadzadeh et al., 2019). A study on the role of fiction on the mentalizing abilities of adolescents has found no differences between written and visual fiction; both are positively related to mentalizing, mainly when fiction is linked to personal engagement directed to happiness (de Mulder et al., 2021). Such data suggest that the psychological process of identification with a character, especially during a long-term narrative exposition through novels, films, and TV series (Muñiz-Velázquez and Delmar, 2021), could contribute to social cognition changes. Less accessible fiction, such as art films with detailed and predictable characterization, shows a broader effect of mentalization abilities measured with the ToM Task (Castano, 2021). These results suggest that the perspective-taking process might be central in the psychological process at stake in the link between fiction and empathy, specifically cognitive empathy.

### Acting empathy

Empathetic understanding is at the core of all drama in the theater (Lada, 1994), but empirical research in this field is relatively new. A recent work (Rathje et al., 2021) has demonstrated that immersing audience members in the story of others could increase their empathy and change their political beliefs. Through three field studies involving the audience at as many different plays focused on people typically marginalized, Rathje et al. (2021) have shown a causality link between theater attendance and empathy, social attitudes, and the intention to donate more to charities unrelated to the shows. Compared to previous eliciting methods, the behavior is actively modified by an empathic response suggesting an integrated top-down/bottom-up mechanism that depends on how the agent lives the experience. Is that whole process increased when the fiction is acted?

Research in the educational sector has investigated forum-theater techniques, inspired by Boal’s conception of the Theater of the Oppressed, as a valuable experience for the social integration of refugee children at school, where students could “put themselves in other people’s shoes” (Day, 2010). Other recent studies tested the same theater technique as a tool for improving interpersonal conflicts at school through proactive participation, critical awareness, and reflexivity (Sappa and Barabasch, 2019). Such a favorable effect in the educational environment has been replicated in the context of real productions such as The Laramie Project, an internationally performed play about social stigma and violence (Corsa, 2020).

New technologies and social media have increased the growth of intergroup empathy sharing. Classical intergroup sharing, such as self- and mutual-help groups, flourished during the pandemic (Sitrin, 2020). Intergroup sharing has overtaken virtual sharing, expanding the possibility of sharing personal experiences. An early study on disability showed how online groups could benefit from mutual problem solving, information sharing, expression of feelings, catharsis, mutual support, and empathy (Finn, 1999). More recently, immersion in virtual video-game worlds has been explored as a possible source of affective ToM development (Bormann and Greitemeyer, 2015). Virtual reality offers a widespread and embodied possibility to elicit the different components of empathy. Empirical research engaging with children in refugee camps shows an increase in the empathy level of participants (Schutte and Stilinović, 2017), and the literature review recently drew a framework for the design of empathetic virtual training (Bertrand et al., 2018). Virtual reality is sometimes viewed as the most promising opportunity for empathy eliciting. However, criticism should be acknowledged on the sustainability of such intervention over time without emotional exhaustion and other side effects (Moroz and Krol, 2018). Limitations should be considered according to the nature of the stressful event in question, such as in the case of a pandemic when social distancing would not allowed group activities. Finally, it should also be acknowledged that some of the interventions proposed in this paragraph may be cost-restrictive and their feasibility depends on access to free time, internet and other similar resources that may limit a large-scale distribution.

## Conclusion

The positive role of empathy in sustaining adherence to public health norms suggests that this is a powerful driver for behavioral response to stressful events such as the current pandemic. Empathy is naturally activated in challenging situations but could decrease over time and lead to emotional exhaustion for individuals exposed to distress. An intertwined top-down and bottom-up psychological mechanism, including cognitive and affective skills, is needed to prevent such negative consequences. Moreover, common ground created by the intersubjective bond between people appears to be fundamental for increasing empathy. Indirect methods of sharing and bearing witness to people’s experiences could be employed: literature and film are of some interest, but more interactive techniques such as theater or virtual reality might be more effective. Further research is needed to establish the effectiveness of such social intervention in eliciting specific components of empathy linked to prosocial behavior during stressful events. It is important to consider the characteristics of adverse events in order to adapt interventions. We live in a time in which empathy is as claimed as it is ephemeral, and we need new opportunities that bring alive our altruistic instinct and intersubjective bond with others.

## Author contributions

All authors listed have made a substantial, direct, and intellectual contribution to the work and approved it for publication.

## Funding

Open access funding provided by Università della Svizzera italiana.

## Conflict of interest

The authors declare that the research was conducted in the absence of any commercial or financial relationships that could be construed as a potential conflict of interest.

## Publisher’s note

All claims expressed in this article are solely those of the authors and do not necessarily represent those of their affiliated organizations, or those of the publisher, the editors and the reviewers. Any product that may be evaluated in this article, or claim that may be made by its manufacturer, is not guaranteed or endorsed by the publisher.

## Footnotes

^1^ http://www.humainologie.com/ourstory/
